# Complete Genome Sequence of Rickettsia asiatica Strain Maytaro1284, a Member of Spotted Fever Group Rickettsiae Isolated from an Ixodes ovatus Tick in Japan

**DOI:** 10.1128/MRA.00886-19

**Published:** 2019-09-12

**Authors:** May June Thu, Yongjin Qiu, Junya Yamagishi, Kodai Kusakisako, Shohei Ogata, Mohamed Abdallah Mohamed Moustafa, Norikazu Isoda, Chihiro Sugimoto, Ken Katakura, Nariaki Nonaka, Ryo Nakao

**Affiliations:** a Laboratory of Parasitology, Graduate School of Veterinary Medicine, Hokkaido University, Hokkaido, Japan; b Hokudai Center for Zoonosis Control in Zambia, School of Veterinary Medicine, The University of Zambia, Lusaka, Zambia; c Division of Collaboration and Education, Research Center for Zoonosis Control, Hokkaido University, Hokkaido, Japan; d Unit of Risk Analysis and Management, Research Center for Zoonosis Control, Hokkaido University, Hokkaido, Japan; University of Maryland School of Medicine

## Abstract

This is the first report of the complete genome sequence of Rickettsia asiatica strain Maytaro1284, isolated from an Ixodes ovatus tick in Japan. The genome contains a 1,344,324-bp circular chromosome and one plasmid of 74,761 bp. There was no outer membrane protein A (*ompA*) gene encoded in the genome.

## ANNOUNCEMENT

The members of the genus *Rickettsia* are Gram-negative obligate intracellular bacteria belonging to the class *Alphaproteobacteria* (1) with 32 designated species divided into four groups, spotted fever (SFG), typhus (TG), transitional (TGR), and ancestral (AG) groups (2). Molecular classification of rickettsial species can be achieved based on the sequences of the 16S rRNA gene, *ompA*, *ompB*, *sca4*, *gltA*, and *htrA* (3).

SFG rickettsiae are mainly associated with ticks, and some are pathogenic for humans. In Japan, six validated SFG rickettsiae, namely, Rickettsia japonica, R. helvetica, R. heilongjiangensis, R. tamurae, R. monacensis, and *R. asiatica*, and several other uncharacterized rickettsial species/genotypes have been detected (4, 5). *R. asiatica* is the only named species of these whose genome sequence is not available.

*R. asiatica*, initially described as a *Rickettsia* strain IO-1^T^, was first isolated from *Ixodes ovatus* ticks collected in Fukushima Prefecture, Japan, in 1993 (6, 7). A recent study revealed that *R. asiatica* had a strong host specificity, where it was detected only in *I. ovatus* (5).

*R. asiatica* strain Maytaro1284 was isolated from an *I. ovatus* tick collected in Yamagata prefecture, Japan (8). In brief, tick homogenate was inoculated into Ixodes scapularis ISE6 cells and incubated at 32°C. The isolate was obtained at 28 days after inoculation without showing any cytopathic effect. *Rickettsia* DNA was purified from infected cells by centrifugation and filtration with a 5.0-μm-pore-size membrane filter. DNA was extracted using a MagAttract HMW DNA kit (Qiagen, Hilden, Germany). An Illumina (Hayward, CA) library was constructed with the Nextera DNA library prep kit and run on an Illumina MiSeq 600-cycle instrument. For PacBio sequencing, genomic DNA was sheared using a G-Tube (Covaris, Woburn, MA) to generate 20-kb fragments. A PacBio library was constructed using the SMRTbell template prep kit 1.0 (Pacific Biosciences, Menlo Park, CA) and sequenced using C4 chemistry on a PacBio RS II instrument.

We acquired 10,353,766 reads with 2,789,655,408 bp and 92,498 reads with 1,954,307,128 bp by MiSeq and PacBio RS II, respectively. The PacBio reads were assembled using PacBio’s Hierarchical Genome Assembly Process (HGAP3, SMRT Analysis 2.3.0) (9), and 3,693 contigs with 8,182,413 bp were obtained. The contigs and raw reads from MiSeq were further assembled using SPAdes 3.11.1 (10), resulting in five contigs of 535,925 bp, 417,525 bp, 311,286 bp, 77,822 bp, and 74,888 bp. The first four contigs were merged into one circular sequence by filling the gaps between contigs using Sanger sequencing. The last contig was manually circularized by eliminating an overlapping end. All software was run with default parameters.

The assembled circular chromosome of 1,344,324 bp had an overall GC content of 32.3%, while one plasmid (pRA1) was 74,761 bp in size with an overall GC content of 33.8%. The average coverages obtained for the chromosome and plasmid genomes were 832× and 1,787×, respectively. Annotation was performed with DFAST (11). The chromosome contains 1,513 protein-coding sequences (CDSs), 3 rRNA genes (5S, 16S, and 23S), and 34 tRNA genes. The plasmid is predicted to contain 194 CDSs.

*R. asiatica* was suspected to lack the *ompA* gene due to the failure of PCR amplification of the gene (7). However, it is also evident that PCR failures of polymorphic genes such as *ompA*, *ompB*, and *sca4* are common issues in the genetic characterization of rickettsiae because of nucleotide mismatches in the primer annealing sites (5, 12). This study confirmed that there is no *ompA* gene in the genome of *R. asiatica*.

### Data availability.

The genome sequences have been deposited at DDBJ/EMBL/GenBank under the accession numbers AP019563 and AP019564, BioProject number PRJDB8064, and BioSample number SAMD00165215. The raw sequencing reads were submitted to the DRA under the accession number DRA008361.
